# Meatoplasty in Canal wall down Surgery: Our Experience and Literature Review

**Published:** 2017-01

**Authors:** Faramarz Memari, Mojtaba Maleki Delarestaghi, Parisa Mir, Mohammad GolMohammadi, Ehsan Shams Koushki

**Affiliations:** 1 *ENT and Head & Neck Research Center, Hazrat Rasoul Akram Hospital, Iran University of Medical Sciences, Tehran, Iran.*; 2 *Department of Otolaryngology and Head and Neck Surgery. Firoozgar Clinical Research Development center, Firoozgar Hospital, Iran University of Medical Sciences, Tehran, Iran.*; 3 *Department of Internal Medicine. Shohada Medical Center, Shahid Beheshti University of Medical Sciences, Tehran, Iran. *

**Keywords:** Canal well down, Meatoplasty, Methods

## Abstract

**Introduction:**

Meatoplasty is the final and essential step in performing effective canal wall down surgery for chronic otitis media. In this article we review some previous techniques and discuss our preferred method.

**Materials and Methods:**

In this observational case series study, we used this technique in 53 patients (28 male and 25 female) between January 2005 and January 2008. Our survey was completed in 31 patients.

**Results:**

Twenty-six patients (83.9%) said their ear appeared normal after the procedure, but five patients (16.1%) complained of some minor change in the shape of their ear. Twenty-nine patients (93.5%) had a completely wide ear canal. The ear canal had some degree of stenosis in two patients (6.5%) post-operatively.

**Conclusion:**

This technique offers good functional and cosmetic results with minimal manipulation and minimal anatomic disruption.

## Introduction

In advanced cases of chronic otitis media or other types of ear disease, an otologist may decide to performed canal wall down (CWD) mastoidectomy surgery. At the end of a CWD mastoidectomy procedure, the external auditory meatus must be widened to provide a dry, self-cleaning ear and to allow for in-office surveillance. Traditionally, a large meatoplasty has been thought to support adequate ventilation and reduce conditions favorable for microbial growth, debris accumulation, and recurrent disease (1). On the other hand, a large meatoplasty will misshape the ear and make it look unnatural. Therefore, there should be a balance between making the meatus large enough for aeration and sufficiently small that it looks “natural”.

Meatoplasty usually is the final step of a CWD mastoidectomy. Meatoplasty provides sufficient enlargement of the external auditory meatus and leaves the patient with a smaller mastoid recess that could readily be cleaned and examined without anatomical restriction. A large meatoplasty supports rapid epithelialization and overall exteriorization of the mastoid bowl size. Failure to perform an adequate meatoplasty may lead to cholesteatoma formation, chronic secretion, and post-operative canal stenosis (2-5). A small meatus after CWD mastoidectomy can cause a permanent problem, and unsatisfactory results are frequently encountered (6).

On the other hand, a large meatoplasty could cause cosmetic deformity of the pinna, especially if the concha cartilage is resected. Meatoplasty procedures can be complicated by stenosis, infection, and pinna deformity. Stenosis of the meatus has the potential to cause accumulation of the cerumen, chronic external otitis, hearing impairment, chronic “wet ear”, and difficulty in examining the ear.

Numerous surgical techniques to prevent and correct meatal stenosis of the ear canal have been described. Meatoplasty techniques include a combination of transposed skin flaps, removal of the conchal cartilage, removal of the cartilage from the tragus or floor of the ear canal, and use of conchomastoid sutures or meatal packing to maintain a large meatal diameter (2-5).

An adequate meatoplasty needs to address three problems: 1) Projection of the anterior edge of the conchal cartilage into the posterior meatus; 2) Excess underlying bone of the posterior bony meatus; 3) Inadequate meatal skin circumference. Inadequate skin coverage predisposes to stenosis leading to wound disruption and infection. 

Since its initial description by Stacke, numerous modifications to the technique of meatoplasty in CWD have been made, primarily by surgeons in the early part of the twentieth century. Most techniques are designed to widen the meatus after radical mastoidectomy in order to create a cavity that can be cleaned easily (4,5,7-14).

Two types of approaches are described for meatoplasty; endaural approach and retro-auricular approach. Some of the most famous approaches described up to 1995 are: Stacke meatoplasty using an endaural approach (14); Korner meatoplasty using an endaural or retro-auricular approach (14); Portmann in a retro-auricular approach (15); Fisch in a retro-auricular approach (16); Mirck in an endaural approach (3); Tong et al. in an endaural approach (17); Fagan et al. in an endaural Z-meatoplasty (18); Gómez-Ullate et al. in an endaural approach (19); Eisenman et al. by a retro-auricular approach (20); Wormald et al. in a retro-auricular approach (21); Suskind et al. combined both the classic Fisch meatoplasty and the Portmann meatoplasty to produce a synergistically advantageous surgical result (22); Rombout and van Rijn used the M-meatoplasty in a retro-auricular approach (23); Raut et al. created an incision endaurally (24); Fisch et al. described an endaural approach meatoplasty for lateral canal stenosis in the absence of mastoidectomy (25); Kim et al. used only a two-stitch technique without incision or resection in post-auricular approaches (26).

## Materials and Methods

**Fig. 1:**
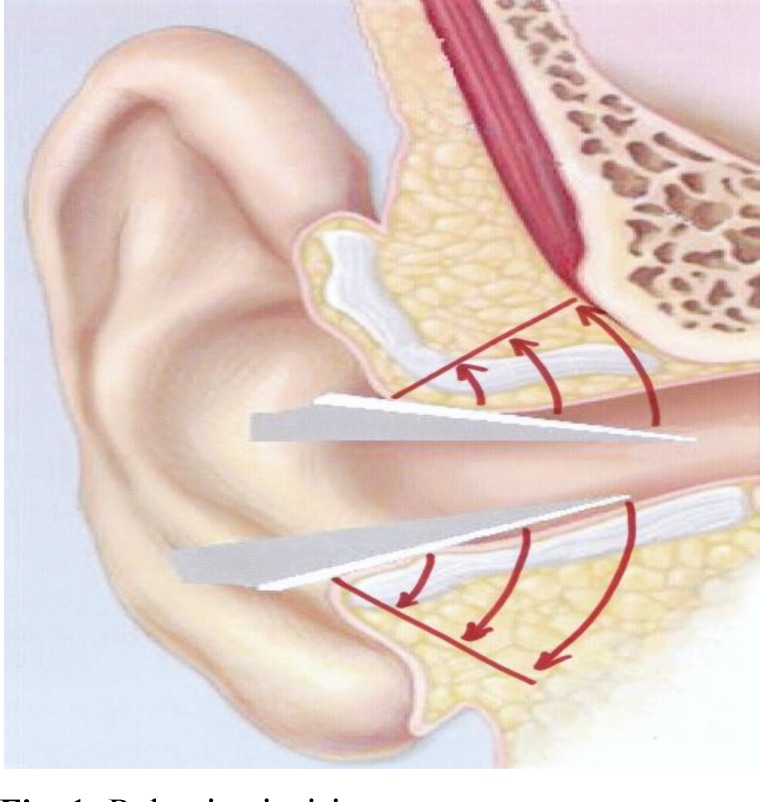
Releasing incisions

In this method, a retro-auricular skin incision is placed 1-cm behind the post-auricular crease. We elevate the skin flap using the cutting mode of monopolar cautery and make an anterior-based U-shaped periosteal incision. The superior periosteal incision is located a few millimeters above the temporalis line, superiorly. After elevation of the periosteal and soft tissue flap, standard CWD mastoidectomy is performed. The mastoid cavity is completely saucerized to reduce the size of the cavity. At the end of the procedure and after placement of a temporalis fascial graft, we incise the canal skin and conchal cartilage at 5 and 12 o’clock, from inside out. At 5 and 12 o’clock, we use a number 11 knife parallel to the skin surface and mastoid cortex from inside towards the lateral part of the meatus to incise the subcutaneous and cartilaginous tissues, but we preserve the skin surface at 5 and 12 o’clock (Fig. 1).

While making these two incisions, care is taken to incise the cartilage and subcutaneous tissue and the skin from medial to the lateral all the way and to stop the incision as soon as we reach the surface skin which is visible from outside. We incise the skin inferiorly at 5 o’clock (intertragic notch) until we reach the lateral border of the concha. 

**Fig 2:**
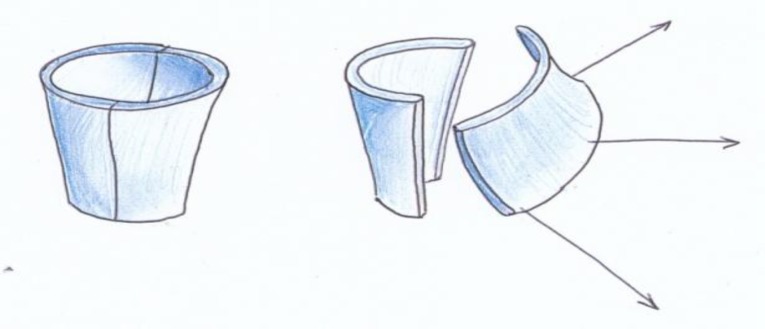
Force direction of sutures

**Fig 3:**
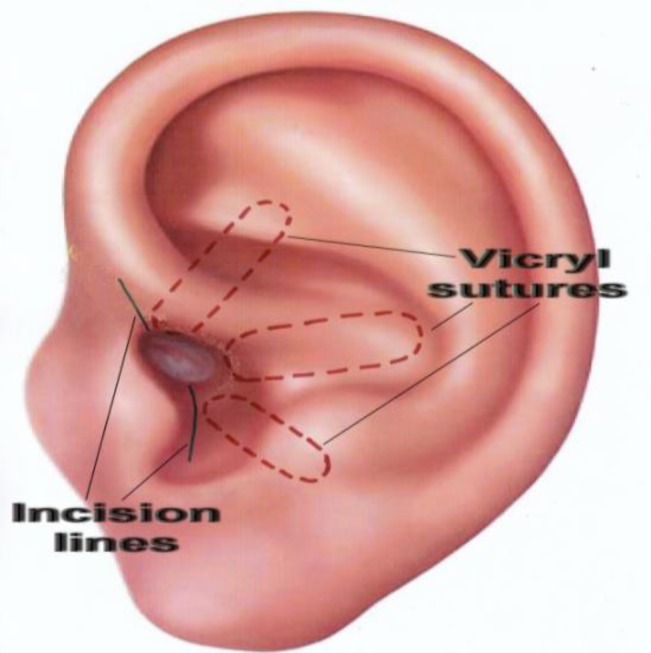
Effect of suture forces on cartilaginous ring

**Fig 4:**
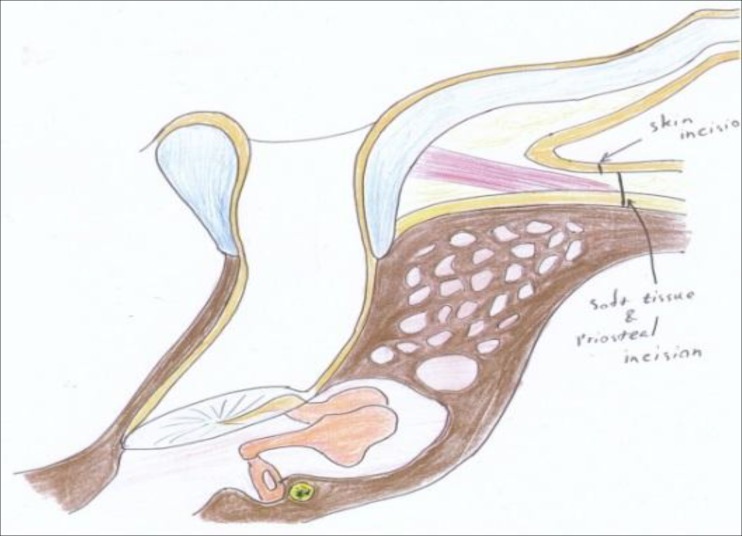
Normal canal and mastoid cavity

**Fig 5:**
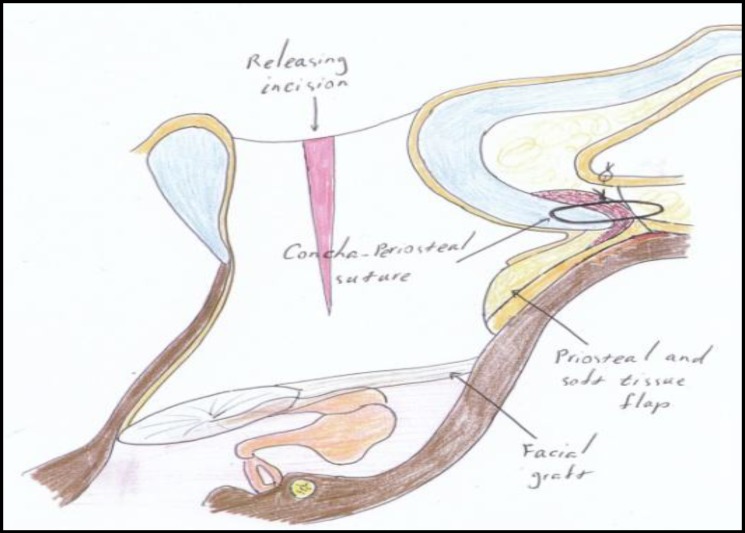
Canal and mastoid cavity after our technique meatoplasty. Note soft tissue and skin covering bare mastoid cavity

Therefore, the cartilaginous ring is broken by these incisions in two places (at 5 and 12 o’clock) to allow the index finger easily to enter the new meatus. Next, three 2-0 vicryl sutures (just lateral to the periosteum as shown in Figures 2 and 3) secure the posterior cartilaginous semi-ring to the posterior edge of our post-auricular incision. By this method, the conchal cartilage is incised at its most anterior inferior part towards the intertragic notch and also at 12 o’clock, and is then pulled back as shown in Figure 3. By releasing the entire cartilaginous ring at its posterior part (including the concha) and moving it backwards, we create a larger ear canal without having to excise the conchal cartilage, and therefore we prevent the cosmetic deformity caused by removing or reshaping the concha. We simply pull the entire posterior cartilaginous ring and concha backwards and create a larger meatal opening. As shown in Figures 4 and 5, the periosteum and soft tissue attached to the concha will obliterate the remaining mastoid cavity and also cover the bare surface of the mastoid cavity. The three vicryl sutures will expand and invert the posterior cartilaginous semi-ring in a radial fashion, as shown in Figure 2.

We use the U-shaped periosteal (Palva) flap to obliterate the mastoid and do not suture the periosteal flap posteriorly. Clearly, we do not close the skin incisions at the 5 and 12 o’clock positions. The two small skin wedges where the cartilage is incised heal and epithelialize spontaneously with minimal granulation tissue formation at these two sites.

Retroauricular skin incision is closed in two layers. We pack the new meatus by a large, compact antibiotic soaked mesh or expandable material (such as Ambrose ear wick or merocele) that remains in place for 14 days. In the post-operative period, prophylactic antibiotic (Cefixime) is prescribed for 10 days.

## Results

**Fig 6:**
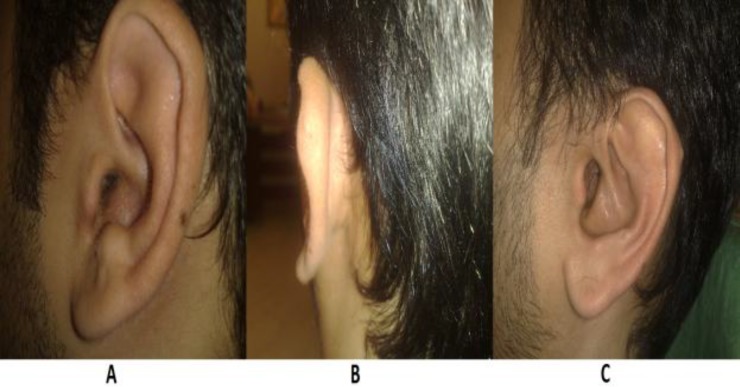
A) pre-operative view; B) post-operative posterior view; C) post-operative lateral view

We used this technique in 53 patients (28 male and 25 female) over the course of 3 years, but our survey was completed in 31 patients. The mean age of patients was 32.8 years (range, 6–59 years; standard deviation (SD), 15.01) and they were followed for a mean period of 30.5 months (range, 5.5–55.8). Twenty-six patients (83.9%) said their ear appeared normal, but five patients (16.1%) complained of some minor change in the shape of their ear. Twenty-nine patients (93.5%) had a completely wide ear canal. Figure 6 shows a pre- and post-operative view of the new meatus after meatoplasty. The ear canal had some degree of stenosis in two patients (6.5%) post-meatoplasty. Only one patient (3.2%) had post-operative granulation tissue in the incision line, which was cauterized with silver nitrate and resolved within a period of 1 week. This patient also had an abnormal ear shape, but we did not find any evidence of perichondritis or canal stenosis. There was only one case (3.2%) of perichondritis, in a patient who had canal stenosis and abnormal shape of the ear. No other complications were noted.

## Discussion

Mirck performed M-meatoplasty in 50 patients. He achieved good results in 42 patients and moderate results in six patients. The only failure occurred in a boy with Down’s syndrome who had undergone bilateral surgery previously and had narrow bony canals (3).

Tong et al. studied 76 patients, 40 of whom underwent a meatoplasty without removal of part of the tragus or use of a sling stitch and 36 who underwent a complete sling stitch meatoplasty as described previously (18). 

Table 1 shows the size of the meatus of patients at least 12 months after surgery. One stitch abscess occurred that was associated with the sling stitch at the post-auricular stab incision, which responded to oral and topical antibiotics. One quarter of the patients who had a sling stitch meatoplasty were noted to have a barely visible cupped ear, which returned to its normal position within 3 months in every case. No long-term cosmetic embarrassment was encountered.

**Table 1:** Meatal size after surgery

**Type**	**Wide** **(>11mm)**	**Adequate** **(8–11mm)**	**Narrow** **(5–8mm)**	**Thigh** **(<5mm)**	**Total number**
Routine Endaural	2	26	9	3	40
Sling stitch meatoplasty	14	22	0	0	36

P<0.1 ( comparing size>8mm and <8mm; Fisher’s Exact Test)

Eisenman et al. achieved a larger meatal area without the need to further extend the posterior conchomeatoplasty, thus avoiding potential cosmetic deformity (21). In the technique used by Wormald et al., the mean largest diameter of the meatus was 10.1 mm and the mean smallest diameter was 8.3 mm; these diameters are comparable with other meatoplasties (22).

Suskind et al. studied 12 CWD mastoidectomy patients. After surgery, all patients had excellent wound healing and achieved large-caliber meatoplasties. The patients were followed-up for 18 months, during which time no complications were noted and none of the cavities required a revision (23).Rombout and van Rijn used the M-meatoplasty technique that was described by Mirck in 199 patients in a retro-auricular approach. The median follow-up of the study population was 11 months. Post-operative wound infections in three patients (1.5%) were the only complications in this study and use of oral ofloxacin resulted in a rapid cure in all three cases, without the final result being affected (24).

Raut et al. reported no cases of perichondritis secondary to cartilage excision in more than 500 cases surgically treated by their method (25).

Similar to Fagan and Ajal, Tunkel used Z-meatoplasty in 24 ears. Twenty-one ears (87.5%) treated with Z-meatoplasty demonstrated healing success and excellent cosmoses without stenosis, during a mean follow-up period of 40 months. Three (12.5%) of the 24 ears that were treated with Z-meatoplasty required additional surgery to revise the meatus during the follow-up period. One of these ears had a post-operative perichondritis that resolved with local care and oral antibiotics, and one developed a keloid at the meatus. All three ears that required revision developed stenosis within 2 months of surgery. These ears are easy to examine and debride with the use of simple otologic tools and an operating microscope (27). Sanna et al. used the Fish method of meatoplasty in 230 cases. Two of these patients (0.8%) developed stenosis of meatoplasty, both of whom required surgical correction. Seven patients (3%) experienced both otorrhea and evidence of granulation tissue in the mastoid cavity that were successfully treated by topical 2% alcoholic solution of boric acid (28). Kim et al. studied 39 ears of patients who had undergone CWD mastoidectomy with the perichondrial posterior fixation technique. The post-operative follow-up duration was 9.5 months on average. All the ears maintained a clean and large external meatus (30). Hovis described one-cut meatoplasty in which (89.3%) patients denied being self-consciousness over the appearance of their ear. Of seven hearing aid users, none reported difficult fitting, discomfort or excessive feedback (1).The results of these different studies and techniques are summarized in Table.2.

**Table 2:** Comparing different methods of ear canal meatoplasty

**Percent of**	**Our results**	**Mirck** ^9^	**Tong** ^18^	**Eisenman** ^21^	**Wormald** ^22^	**Suskind** ^23^	**Rombout** ^24^	**Raut** ^25^	**Tunkel** ^27^	**Sana** ^28^	**Kim** ^29^
Granulation tissue	3.2	-	-	-	-	0	-	-	4.1	3	-
Stenosis	6.5	4	0	-	0	0	-	-		0.8	0
Perichondritis	3.2	-	2.7	-	-	0	1.5	0	4.1	-	-
Need for revision	0	-	-	-	-	0	-	-	12.5	-	-
Cosmetic satisfaction	84	-	100	100	-	-	-	-	88	-	-

In our method, we use 5 and 12 o’clock incisions to disrupt the cartilaginous ring. Instead of resecting the conchal cartilage that could result in cosmetic deformity of the pinna, we use concha-periosteal absorbable sutures (as done by Kim et al) and meatal packing to keep the disrupted canal ring open. Since the cartilaginous ring is disrupted in two places in this method, the new meatus will remain open using the above mentioned sutures. The posterior skin flap and subcutaneous soft tissues and periosteum will cover and fill the bare mastoid cavity, facilitate epithelialization of the cavity and shorten post-operative recovery time.

Although Almario advocated not leaving the cartilage exposed to prevent perichondritis, current powerful antibiotics enables us to avoid perichondritis even though we leave the incised cartilage at 12 and 5 o’clock. The two small skin wedges where the cartilage is incised will heal and epithelialize spontaneously with minimal granulation tissue formation at these two sites.

## Conclusions

This article represents the result of the learning curve in 53 patients. Since this article was written, the senior author has continued using this method of meatoplasty and observed that after the learning curve was over and in the following 200 cases, the only discomfort/ complication noted in a few cases was minimal granulation tissue formation in the area that the cartilage was incised.Finally we conclude that this technique offers good functional and cosmetic results with minimal manipulation and minimal anatomic disruption.
